# Highly efficient conversion of xylose to ethanol without glucose repression by newly isolated thermotolerant *Spathaspora passalidarum* CMUWF1–2

**DOI:** 10.1186/s12866-018-1218-4

**Published:** 2018-07-13

**Authors:** Nadchanok Rodrussamee, Pachara Sattayawat, Mamoru Yamada

**Affiliations:** 1 Department of Biology, Faculty of Science, Chiang Mai University, Chiang Mai, 50200 Thailand; 2 Center of Excellence in Bioresources for Agriculture, Industry and Medicine, Chiang Mai University, Chiang Mai, 50200 Thailand; 3 Life Science, Graduate School of Science and Technology for Innovation, Yamaguchi University, Ube, 755-8505 Japan; 4 Department of Biological Chemistry, Faculty of Agriculture, Yamaguchi University, Yamaguchi, 753-8515 Japan; 5 Research Center for Thermotolerant Microbial Resources, Yamaguchi University, Yamaguchi, 753-8315 Japan

**Keywords:** *Spathaspora passalidarum*, Bioethanol, Glucose repression, Thermotolerant yeast, Lignocellulosic biomass

## Abstract

**Background:**

Efficient bioconversion of lignocellulosic biomass to bioethanol is one of key challenges in the situation of increasing bioethanol demand. The ethanologenic microbes for such conversion are required to possess abilities of utilization of various sugars including xylose and arabinose in lignocellulosic biomass. As required additional characteristics, there are a weak or no glucose repression that allows cells to simultaneously utilize various sugars together with glucose and thermotolerance for fermentation at high temperatures, which has several advantages including reduction of cooling cost. *Spathaspora passalidarum* ATCC MYA-4345, a type strains, isolated previously have mainly of these abilities or characteristics but its thermotolerance is not so strong and its glucose repression on xylose utilization is revealed.

**Results:**

Newly isolated *S. passalidarum* CMUWF1–2 was found to have a high ability to produce ethanol from various sugars included in lignocellulosic biomass at high temperatures. The strain achieved ethanol yields of 0.43 g, 0.40 g and 0.20 g ethanol/g xylose at 30 °C, 37 °C and 40 °C, respectively. Interestingly, no significant glucose repression was observed in experiments with mixed sugars, being consistent with the strong resistance to 2-deoxyglucose, and antimycin A showed no effect on its growth in xylose medium. Moreover, the strain was tolerant to glucose and ethanol at concentrations up to 35.0% (*w*/*v*) and 8.0% (*v*/v), respectively.

**Conclusions:**

*S. passalidarum* CMUWF1–2 was shown to achieve efficient production of ethanol from various sugars and a high ethanol yield from xylose with little accumulation of xylitol. The strain also exhibited stress-resistance including thermotolerance and no detectable glucose repression as beneficial characteristics. Therefore, *S. passalidarum* CMUWF1–2 has remarkable potential for conversion of lignocellulosic biomass to bioethanol.

**Electronic supplementary material:**

The online version of this article (10.1186/s12866-018-1218-4) contains supplementary material, which is available to authorized users.

## Background

Bioethanol obtained from lignocellulosic biomass such as agricultural residues has received increasing attention as an alternative to petroleum-based fuels because of the problem of global warming and the necessity to reduce emission of carbon dioxide [1]. Utilization of lignocellulosic biomass, which is the largest source of fermentable sugars for bioconversion to ethanol fuel, causes no problem regarding the issue of fuel-food conflicts unlike the use of corn or sugar cane [2].

Lignocellulose consists of three main parts, cellulose, hemicellulose and lignin, with different relative proportions of the three parts depending on the material source [3]. Cellulose is a homopolymer of a β-1,4-linked glucose unit. The linear chains of cellulose form highly crystalline microfibrils. Hemicellulose is a xylan backbone consisting of β-1,4-linked xylose monomers to which substituents and saccharides are attached [4]. To improve the economics for ethanol production from lignocellulose, all of the sugars present in lignocellulose hydrolysates must be effectively converted into ethanol. However, one problem is the low conversion efficiency of pentose sugars in fermenting microbes, and xylose, which is the most abundant pentose sugar in hemicellulose, cannot be utilized by wild-type *Saccharomyces cerevisiae*, which has long been used for ethanol production in industries [5].

Additionally, ethanol production with lignocellulose as a starting material is a complicated process. Generally, the process consists of at least three steps: pretreatment, hydrolysis and fermentation. After the pretreatment and hydrolysis steps, sugars are released from lignocellulose and utilized for fermentation [6]. Microorganisms that can convert all types of sugars (glucose, mannose, galactose, xylose and arabinose) in cellulose and hemicellulose hydrolysates and have the ability to efficiently metabolize mixed sugar substrates are prerequisite for making the lignocellulosic ethanol processes as an economically competitive processes [7]. Ideal microorganisms should thus not only display broad substrate specificity and high ethanol yield and productivity but also have other relevant abilities such as substrate tolerance, ethanol tolerance, temperature tolerance and tolerance to inhibitors present in hydrolysates [8].

There are many types of yeast that can assimilate xylose, but only a few can ferment xylose. Normally, native xylose-fermenting yeasts such as *Scheffersomyces* (*Pichia*) *stipitis* [9], *Scheffersomyces* (*Candida*) *shehatae* [10], *Pachysolen tannophilus* [11] and *Kluyveromyces marxianus* [12, 13] can ferment both glucose and xylose. However, the rate of ethanol production and yield from xylose when using those yeasts are usually lower than those from the main hexose sugars, mannose and glucose. Besides, there are crucial problems for yeast cells or for yield in the ethanol fermentation process, including presence of inhibitors in lignocellulose hydrolysate, less tolerance of ethanol concentration, exhibit of glucose repression and formation of by-products [14].

*Spathaspora passalidarum* is a wood-boring beetle-associated yeast [15]. It can ferment xylose under both aerobic and anaerobic conditions with a higher efficiency than that of *S. stipitis* [16]. This strain is capable of co-fermenting xylose and cellobiose in the presence of glucose under oxygen-limiting conditions [17]. Moreover, its complete genome sequence has been determined [18], and it is therefore useful for research in molecular biology. These interesting properties suggest that *S. passalidarum* is a promising candidate for industrial bioethanol production from lignocellulosic biomass.

In this study, a new strain, *S. passalidarum* CMUWF1–2, was isolated from soil in Chiang Mai, Thailand. This report is the first report of a species that can grow and co-ferment glucose and one of sugars, mannose, galactose, xylose and arabinose, at high temperatures. We also examined the effect of antimycin A on xylose utilization for testing the requirement of respiratory activity for maintenance of cofactor balance and various stresses on the growth of this strain.

## Results

### Isolation, analysis of growth and ethanol production of xylose-fermenting yeasts and their identification

**Fig. 1:**
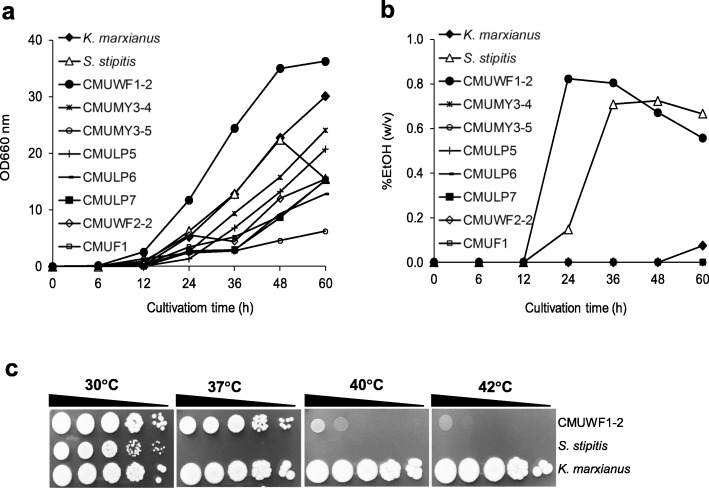
Isolation, characterization and identification of xylose-fermenting yeasts. Growth (**a**) and ethanol production ability (**b**) of isolated xylose-utilizing yeasts were examined in YPXyl medium at 30 °C and 160 rpm. Data presented are averages of two independent experiments. Symbols for the data are as follows: , *K. marxianus*; , *S. stipitis*; , CMUWF1–2; , CMUMY3–4; , CMUMY3–5; , CMULP5; , CMULP6; , CMULP7; , CMUWF2–2 and , CMUF1. Growth ability of CMUWF1–2 was tested on YPXyl plates at various temperatures (**c**). Data were reproduced by two independent experiments

Thirteen isolates of xylose-assimilating yeasts were acquired from natural samples in Chiang Mai Province or Chiang Rai Province, Thailand (Additional file 1: Table S1). From eight of all isolates, which could grow well at 37 °C on YPXyl plates, only CMUWF1–2, which was isolated from soil in Mae Taeng District, Chiang Mai Province, showed the highest growth rate on YPXyl medium (Fig. 1a). The growth rate of CMUWF1–2 was also 1.3-times higher than those of *S. stipitis* and *K. marxianus.* From initial substrate concentration of 20 g/l xylose, CMUWF1–2 gave the highest ethanol concentration, 8.23 g/l, after 24 h, which was larger than those of *S. stipitis* and *K. marxianus*, which gave the highest ethanol concentrations, 7.35 g/l and 0.75 g/l, at 48 h and 60 h, respectively (Fig. 1b). The highest ethanol production of CMUWF1–2 was 11- times and 1.1-times higher than those of *K. marxianus* and *S. stipitis*, respectively (Fig. 1b). Hence, CMUWF1–2 was the best xylose-fermenting yeast. Moreover, the growth ability of CMUWF1–2 was examined at various temperatures in comparison with those of *S. stipitis* and *K. marxianus*, which are widely known for their abilities to rapidly ferment xylose [19] and to assimilate xylose at high temperatures [13], respectively (Fig. 1c). *K. marxianus* was able to grow at all tested temperatures. Growth of CMUWF1–2 was more obvious at 30°C and 37°C but it could grow at high temperatures, even at 42°C, exhibiting the property of thermotolerance. On the other hand, *S. stipitis* could grow only at 30°C.

CMUWF1–2 was further subjected to identification by sequencing of the D1/D2 domain of the large subunit rRNA gene. The determined nucleotide sequence was deposited in DNA Data Bank of Japan (DDBJ) as LC062703. The D1/D2 domain sequence of CMUWF1–2 was 99% identical with 1 nucleotide difference to that of *S****.*** *passalidarum* strain ATCC MYA-4345 (FJ614681). A phylogenetic tree was constructed on the basis of sequences of the D1/D2 domain in the large-subunit rDNA gene. It showed that CMUWF1–2 was located in the same position as *S****.*** *passalidarum* (Additional file 2: Figure S1). According to the guide of Kurtzman and Robnett [20], yeast strains with 0–3 nucleotide differences are conspecific or sister species. Therefore, CMUWF1–2 was concluded to be *S***.** *passalidarum*.

### Cell growth and ethanol production in YP medium containing a single sugar at different temperatures

**Fig. 2:**
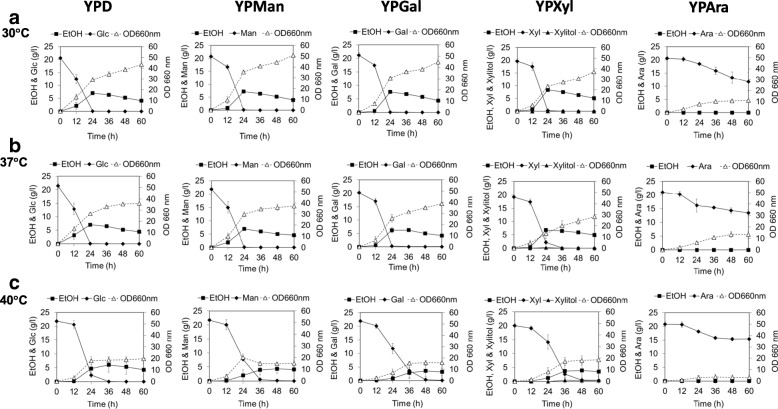
Growth and metabolite profiles of *S. passalidarum* CMUWF1–2 grown in YP medium containing 20 g/l of glucose (YPD), mannose (YPMan), galactose (YPGal), xylose (YPXyl) or arabinose (YPAra) at 30 °C (**a**), 37 °C (**b**) and 40 °C (**c**). Error bars represent ± SD for three independent experiments. Symbols for the data are as follows: , EtOH; , each of sugars (Glc, Man, Gal, Xyl and Ara); , Xylitol; , biomass

The ability of CMUWF1–2 to utilize each sugar that was presented in lignocellulosic biomass and produce ethanol from each sugars was examined. Cells were grown in YP medium containing Glc, Man, Gal, Xyl or Ara at different temperatures, and cell growth and concentrations of sugars and ethanol in the medium were monitored (Fig. 2; Table 1; Additional file 3: Table S2).

**Table 1:** Parameters in YP medium containing a single sugar at various temperatures under a shaking condition (160 rpm)

Medium	Temp (°C)	Max. EtOH (g/l)	Max. Y_p/s_ (g/g)	Max. μ_x/s_ (h^− 1^)	Max. γ_s_ (g/l⋅h)
YPD	30	6.98(24) ± 0.23	0.34 ± 0.03	1.36(24) ± 0.28	1.03(24) ± 0.15
	37	7.02(24) ± 0.58	0.33 ± 0.01	1.12(12) ± 0.09	1.07(24) ± 0.18
	40	6.00(36) ± 2.66	0.28 ± 0.13	1.21(24) ± 0.28	1.53(24) ± 0.13
YPMan	30	7.14(24) ± 0.16	0.35 ± 0.01	2.19(24) ± 0.11	1.38(24) ± 0.08
	37	6.95(24) ± 0.13	0.32 ± 0.01	1.65(24) ± 0.06	1.24(24) ± 0.19
	40	4.33(48) ± 1.54	0.20 ± 0.07	1.35(24) ± 0.36	1.03(24) ± 0.12
YPGal	30	7.57(24) ± 0.26	0.36 ± 0.02	1.91(24) ± 0.10	1.45(24) ± 0.02
	37	6.21(36) ± 0.41	0.31 ± 0.04	1.63(24) ± 0.06	1.40(24) ± 0.09
	40	3.56(48) ± 1.99	0.17 ± 0.09	0.69(36) ± 0.34	0.69(24) ± 0.18
YPXyl	30	8.36(24) ± 0.22	0.43 ± 0.03	1.46(24) ± 0.14	1.44(24) ± 0.11
	37	6.77(24) ± 0.38	0.40 ± 0.04	0.70(24) ± 0.26	1.27(24) ± 0.12
	40	3.90(48) ± 2.46	0.20 ± 0.12	0.75(36) ± 0.31	0.96(36) ± 0.16
YPAra	30	0.00	0.00	0.37(24) ± 0.12	0.22(48) ± 0.08
	37	0.00	0.00	0.38(36) ± 0.02	0.34(24) ± 0.11
	40	0.00	0.00	0.17(24) ± 0.12	0.21(24) ± 0.09

Values in parentheses represent cultivation times that required for reaching the maximum values; ±, SD from three independent experiments

*Max.Y*_*p/s*_ Maximum ethanol yield, *Max.μ*_*x/s*_ Maximum growth rate, *Max.γ*_*s*_ Maximum sugar utilization rate

In all tested sugars at all tested temperatures, cell growth was observed as the amount of the sugar decreased, indicating that the strain can utilize these sugars for growth even at high temperatures. CMUWF1–2 grew well and showed ethanol accumulation in all sugars except for Ara. No long lag phase was observed in any of the sugars. In YPXyl, very small amounts of xylitol, 0.03, 0.19 and 0.43 g/l, were detected at 30 °C, 37 °C and 40 °C, respectively.

At 30 °C (Fig. 2a and Table 1), highest levels of ethanol production from Glc, Man, Gal and Xyl were observed at 24 h. The highest maximum ethanol yield was found in YPXyl, and it was calculated to be 0.43 g ethanol/g xylose, corresponding to 84% of the theoretical yield. The yield was 1.3-, 1.2- and 1.2-times higher than those in YPD, YPMan and YPGal, respectively. All of the sugars were completely consumed within 24 h except for Ara, which was not completely utilized within 60 h.

At 37 °C (Fig. 2b and Table 1), the maximum ethanol yield was slightly reduced from those at 30 °C in all sugars and was also the highest in YPXyl, 0.40 g/g. The maximum specific growth rates were slightly different in all sugars from those at 30 °C. Utilization of Glc, Man and Gal utilizations was completed at 24 h as was found in the experiments at 30 °C, whereas Xyl utilization was delayed compared to that at 30 °C and was completed at 36 h. Ara still remained in the medium at 60 h.

At 40 °C (Fig. 2c and Table 1), the maximum ethanol yield, maximum specific growth rate and maximum specific sugar utilization rate for all sugars were clearly reduced and there was a delay for reaching each maximum value. A comparison of these values for each sugar with those at 30 °C showed that they were 1–3-times reduced, especially in YPGal, YPXyl and YPAra.

### Cell growth and ethanol production in YP medium including mixed sugars with glucose at different temperatures

**Fig. 3:**
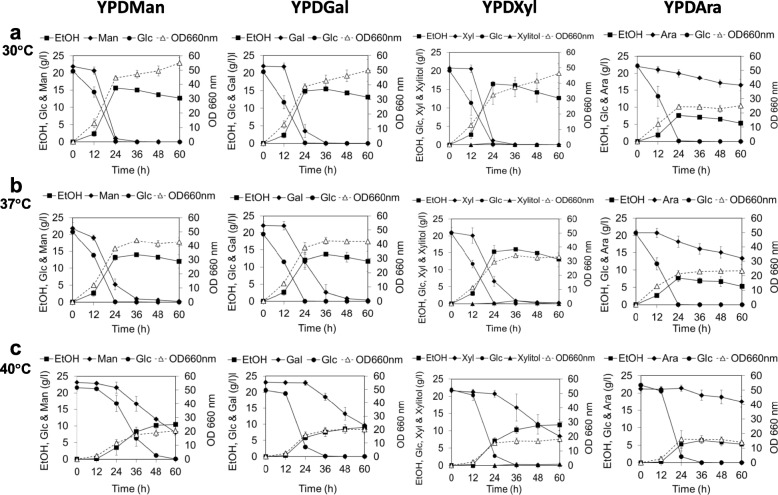
Growth and metabolite profiles of *S. passalidarum* CMUWF1–2 grown in YP medium containing 20 g/l glucose with 20 g/l of mannose (YPDMan), galactose (YPDGal), xylose (YPDXyl) or arabinose (YPDAra) at 30 °C (**a**), 37 °C (**b**) and 40 °C (**c**). Error bars represent ± SD for three independent experiments. Symbols for the data are as follows: , EtOH;  each of sugars (Man, Gal, Xyl and Ara); , Xylitol; , Glc; , biomass

The capability of *S. passalidarum* ATCC MYA-4345 for co-fermenting glucose, xylose and cellobiose and consuming these sugars in anaerobic and aerobic conditions has been reported [16, 17]. However, the glucose effect of this species on assimilation of other sugars that are present in lignocellulose has never been tested. We thus performed experiments to examine the effect of glucose on utilization of other sugars at different temperatures (Fig. 3; Table 2; Additional file 4: Table S3).

**Table 2:** Parameters in YP medium containing mixed sugars with Glc at various temperatures under a shaking condition (160 rpm)

Medium	Temp (°C)	Max. EtOH (g/l)	Max. Y_p/s_ (g/g)	Max. μ_x/s_ (h^−1^)	Max. γ_s_ (g/l⋅h)	
YPDMan	30	15.6(24) ± 0.41	0.38 ± 0.03	2.65(24) ± 0.19	Glc Man	1.19(24) ± 0.12 1.64(24) ± 0.14
	37	14.0(36) ± 0.37	0.35 ± 0.02	2.17(24) ± 0.05	Glc Man	1.14(24) ± 0.06 1.16(24) ± 0.17
	40	10.4(60) ± 0.54	0.28 ± 0.02	0.77(24) ± 0.18	Glc Man	0.88(36) ± 0.05 0.41(36) ± 0.14
YPDGal	30	15.5(36) ± 0.27	0.37 ± 0.01	2.16(24) ± 0.30	Glc Gal	0.97(24) ± 0.15 1.53(24) ± 0.23
	37	13.7(36) ± 0.15	0.35 ± 0.02	2.10(24) ± 0.31	Glc Gal	0.96(24) ± 0.05 0.88(24) ± 0.25
	40	9.08(60) ± 1.58	0.27 ± 0.03	1.14(24) ± 0.10	Glc Gal	1.39(24) ± 0.05 0.44(48) ± 0.08
YPDXyl	30	16.5(24) ± 0.75	0.42 ± 0.03	1.64(24) ± 0.28	Glc Xyl	0.94(24) ± 0.27 1.61(24) ± 0.09
	37	16.0(36) ± 0.44	0.39 ± 0.02	1.50(24) ± 0.07	Glc Xyl	0.97(24) ± 0.12 1.13(24) ± 0.29
	40	11.7(60) ± 1.57	0.33 ± 0.04	1.09(24) ± 0.05	Glc Xyl	1.46(24) ± 0.15 0.40(48) ± 0.12
YPDAra	30	7.61(24) ± 0.57	0.32 ± 0.03	1.02(12) ± 0.35	Glc Ara	1.08(24) ± 0.24 0.09(24) ± 0.12
	37	7.71 (24) ± 1.04	0.33 ± 0.04	1.07(12) ± 0.04	Glc Ara	0.98(24) ± 0.15 0.22(24) ± 0.03
	40	6.38 (36) ± 0.56	0.27 ± 0.00	1.12(24) ± 0.36	Glc Ara	1.58(24) ± 0.15 0.17(36) ± 0.13

Values in parentheses represent cultivation times that required for reaching the maximum values; ±,  SD from three independent experiments

*Max.Y*_*p/s*_ Maximum ethanol yield, *Max.μ*_*x/s*_ Maximum growth rate, *Max.γ*_*s*_ Maximum sugar utilization rate

Patterns of Glc utilization in YPDGal, YPDXyl and YPDAra were similar to those in YPD at 30 °C, 37 °C and 40 °C. However, utilization of glucose in YPDMan did not follow these pattern, which both glucose and mannose were consumed slower when combined together (YPDMan) that was not seen for the other combinations (YPDXyl, YPDGal and YPDAra) (Figs. 2 and 3).

Glc was simultaneously co-utilized with each other sugar at different utilization rates. In the presence of Glc, the utilization rate of other sugars, especially at 12 h, was low and the consumption was delayed compared to that in the absence of Glc, and this tendency was more evident at higher temperatures (Figs. 2 and 3).

At 30 °C (Fig. 3a and Table 2), the ethanol production level in all cases was highest at 24 h except for YPDGal. The maximum ethanol yield was highest in YPDXyl, 0.42 g/g. The maximum growth rate was highest in YPDMan, 2.65 ± 0.19 h^− 1^. The utilization rate of Glc at 12 h was 5, 72, 36 and 9-times higher than those of Man, Gal, Xyl and Ara, respectively (Additional file 4: Table S3). Glc, Man and Xyl were almost completely consumed within 24 h, while Gal was completely consumed at 36 h. However, the consumption of Ara was not completed within 60 h.

At 37 °C (Fig. 3b and Table 2), growth and maximum growth rates were slightly reduced in all cases except for YPDAra. The maximum utilization rates of Man, Gal and Xyl from 12 to 60 h were lower than those at 30 °C, except for that of Ara. A large amount of Ara remained even after 60 h. The peaks of ethanol production in YPDMan and YPDXyl were shifted to 36 h, while those in YPDGal and YPDAra were not changed from those at 30 °C. The maximum ethanol yield was highest in YPDXyl, 0.39 g/g, which was not greatly different from that in YPXyl at the same temperature (0.40 g/g).

At 40 °C (Fig. 3c and Table 2), the utilization of other sugars in the presence of Glc was retarded and their consumption was not completed within 60 h. The highest ethanol production levels in all sugars were observed at 60 h expect for Ara. On the other hand, time required to reach the highest level of ethanol production in YPDAra was very short (36 h), possibly due to ethanol production only from Glc but not from Ara (see Fig. 2). The maximum ethanol yield was highest in YPDXyl, 0.33 g/g, which was 1.7-times higher than that of YPXyl at 40 °C.

Taken together, the results suggested that *S. passalidarum* CMUWF1–2 has high conversion efficiency in ethanol production from both hexose and pentose sugars even at a high temperature and performs it without significant glucose repression and with very little accumulation of xylitol when Xyl is used as a carbon source. Further detailed experiments are required to confirm whether this strain has glucose repression or not.

### Effects of different concentrations of 2-deoxyglucose (2-DOG) as a glucose analogue

Since it was assumed that the strains to be tested would exhibit different sensitivities to 2-DOG as a non-metabolizable glucose analogue, which is known to cause glucose repression [21], effects of different concentrations of 2-DOG were examined. YPXyl plates containing various concentrations, 0.0, 0.01, 0.05, 0.10 and 0.20%, of 2-DOG were used. As shown in Additional file 5: Figure S2, *S. passalidarum* CMUWF1–2 was found to grow well at all concentrations of 2-DOG, though slight growth inhibition in colony size was observed at 2-DOG concentrations of more than 0.10%. On the other hand, strong growth inhibition in *K. marxianus* and *S. stipitis* was observed at concentrations of more than 0.01 and 0.1%, respectively.

### Effect of 2-DOG on growth in YP media containing other sugars

**Fig. 4:**
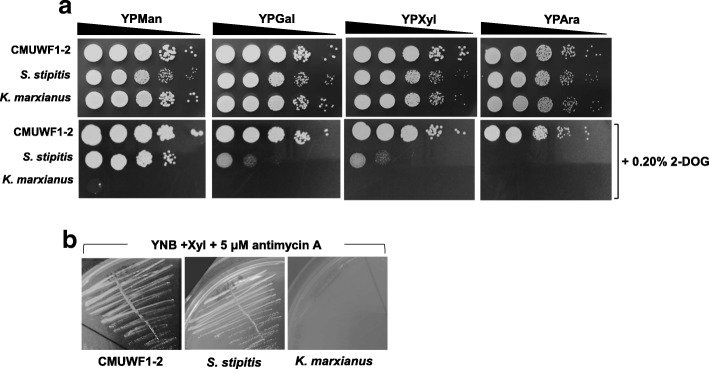
Effects of 2-deoxyglucose (2-DOG) and antimycin A on *S. passalidarum* CMUWF1–2. Effects of 2-DOG and antimycin A on *S. passalidarum* CMUWF1–2 were compared with those on *K. marxianus* and *S. stipitis*. Effects of 2-DOG were examined on YPMan, YPGal, YPXyl, and YPAra plates with or without 0.2% 2-DOG (**a**). Effects of antimycin A were examined on YNB plates containing 2% Xyl and 5 μM antimycin A (**b**). All data were reproduced by two independent experiments

In the case of *S. passalidarum* ATCC MYA-4345, glucose repression on Xyl has been reported [16], but glucose repression on other sugars has not been reported yet. To investigate the effect of 2-DOG on growth in YP media containing other sugars that are present in lignocellulose, YPMan, YPGal, YPXyl and YPAra plates with and without 0.01% or 0.20% 2-DOG were used. In the presence of 0.01% 2-DOG, growth inhibition was clearly observed in *K. marxianus* on YPGal, YPXyl and YPAra plates, while no growth inhibition on other sugars in *S. stipitis* and *S. passalidarum* CMUWF1–2 was found (Additional file 6: Figure S3). In all plates containing 0.20% 2-DOG, *S. stipitis* clearly showed growth inhibition on Gal, Xyl and Ara and no growth of *K. marxianus* was observed. While, CMUWF1–2 exhibited no effect of 2-DOG on any sugars (Fig. 4a). These results are consistent with those shown in Fig. 3. These findings suggest that *K. marxianus* and *S. stipitis* suffer from glucose repression on Gal, Xyl and Ara but that *S. passalidarum* CMUWF1–2 can utilize these sugars without glucose repression.

### Effect of antimycin A on growth in YPXyl medium

Antimycin A is known to prevent oxidative phosphorylation by blocking respiratory activity through inhibition of complex III in mitochondria [22]. In the presence of antimycin A, the cytoplasmic redox balance is maintained by coupling the oxidation of glycolytic NADH to the reduction of acetaldehyde to ethanol [23].

To examine whether xylose utilization in *S. passalidarum* CMUWF1–2 requires respiratory activity and/or oxidative phosphorylation or not, this strain as well as *K. marxianus* and *S. stipitis* were grown on YNB plates containing 2% Xyl and 5 μM antimycin A (Fig. 4b). *S. passalidarum* CMUWF1–2 and *S. stipitis* grew well, but *K. marxianus* could not grow. These results suggest that *S. passalidarum* CMUWF1–2 and *S. stipitis* require neither respiratory activity nor oxidative phosphorylation for Xyl utilization but that they are both essential for *K. marxianus* to grow on Xyl.

### Glucose tolerance and ethanol tolerance of *S. passalidarum* CMUWF1–2

**Fig. 5:**
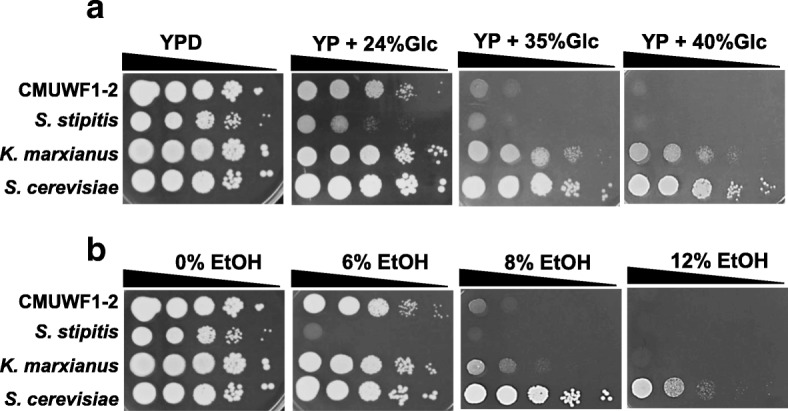
Glucose tolerance and ethanol tolerance of *S. passalidarum* CMUWF1–2. Glucose tolerance (**a**) and ethanol tolerance (**b**) of *S. passalidarum* CMUWF1–2 were compared with those of *K. marxianus* and *S. stipitis*. Data were reproduced by two independent experiments

Glucose tolerance and ethanol tolerance are crucial traits for efficient ethanol fermentation. *S. passalidarm* CMUWF1–2 was thus subjected to a high glucose concentration and a high ethanol concentration (Fig. 5a; Additional file 7: Figure S4a). In experiments for glucose tolerance, *S. passalidarm* CMUWF1–2 was found to grow with concentrations of Glc up to 35% (*w*/*v*), and the growth was slightly higher than that of *S. stipitis* at the same concentration of Glc. On the other hand, *K. marxianus* and *S. cerevisiae* could grow at concentrations up to 40% (w/v). For ethanol tolerance (Fig. 5b; Additional file 7: Figure S4b), *S. passalidarm* CMUWF1–2 could tolerate 8% ethanol (*v*/v), which was nearly equivalent to the tolerance of *K. marxianus* but less than that of *S. cerevisiae*, which grew in the presence of 12% (v/v) ethanol. Nevertheless, *S. passalidarm* CMUWF1–2 was more resistant to ethanol than was *S. stipitis*, which could not grow at an ethanol concentration of more than 6% (v/v).

## Discussion

A type strain of *S. passalidarum*, ATCC MYA-4345 (CBS 10155 = NRRL Y27907), has been isolated from the gut of *Odontotaenius disjunctus* (wood-boring beetle) in the eastern part of the United States [15]. Other strains of *S. passalidarum* have been isolated from rotting wood collected, wood-boring beetles and log galleries sampled in Brazilian Amazonian Forest [24, 25] and rotten wood in central China [26]. *S. passalidarum* CMUWF1–2, however, was firstly isolated from soil and firstly found in Southeast Asia.

*S. passalidarum* CMUWF1–2 was able to utilize all of sugars present in lignocellulosic biomass even at high temperature. However, at 40 °C, the reduction of maximum ethanol yield, maximum specific growth rate and maximum specific sugar utilization rate were found in YPGal, YPXyl and YPAra more than YPD and YPMan. These might be due to the reduction of ATP levels at high temperatures [27], which may affect the uptake of sugars, since many sugar transporters, especially for Xyl in xylose-fermenting yeast, require ATP for generating a proton motive force that drives high-affinity sugar-proton symporters [28, 29].

In this study, *S. passalidarum* CMUWF1–2 was found to have different characteristics from those of *S. passalidarum* ATCC MYA-4345 that were previously reported [15]. ATCC MYA-4345 is unable to utilize L-Ara for growth, while CMUWF1–2 was shown to utilize L-Ara even at high temperatures. CMUWF1–2 was able to grow at temperatures up to 42 °C, but ATCC MYA-4345 does not seem to grow at temperatures over 40 °C [15]. The dissimilar characteristics of the two stains might be due to the original source of isolation. ATCC MYA-4345 was originally isolated from the passalid beetle midgut, which is hypothesized to be an oxygen-limited environment [15], while CMUWF1–2 was originally isolated from soil, which is exposed to oxygen. Moreover, CMUWF1–2 was isolated from Thailand, a tropical country, and it might therefore have adapted to tropical, high-temperature environments. CMUWF1–2 may thus be the most thermotolerant strain in *S. passalidarum* strains isolated so far. Notably, this is the first report of characterization of growth and fermentation at a relatively high temperature as a species that has been characterized at 25–32 °C [16, 17, 24, 25, 30–33]. A summary of Xyl fermentation of *S. passalidarum* CMUWF1–2 in this study and other strains, which were performed at temperature between 25 and 30 °C is shown in Additional files 8: Table S4.

There were researches that studied cofermentation of glucose with xylose, and/or cellobiose in *S. passalidarm* ATCC MYA-4345 [16, 17, 34, 35]. However, they did not perform cofermetation of glucose with other sugars such as mannose, galactose or arabinose. Among these researches, there was only one experiment from Hou (2012) that he mentioned about glucose repression on xylose in *S. passalidarm* ATCC MYA-4345, especially in the presence of 2-DOG in xylose containing medium [16]. Thus, for testing glucose repression in CMUWF1–2, Glc was added together with each other sugars− Man, Gal, Xyl and Ara and for confirmation of glucose repression on other sugars in CMUWF1–2, 2-DOG was added. The effect of 2-DOG on xylose medium between CMUWF1–2 and ATCC MYA-4345 was different. Xyl consumption in ATCC MYA-4345 is partially inhibited by the presence of 0.10% 2-DOG under aerobic conditions and thus the complete utilization of Xyl is greatly delayed [16], while 0–0.20% 2-DOG was no effect to growth in CMUWF1–2 (Additional file 5: Figure S2).

In YPDMan, utilization rates of Glc and Man were lower than those of each sugar in YPD and YPMan (Figs. 2 and 3). This phenomenon was not from glucose repression on Man, however this would be due to competition of the two sugars in uptake by the same hexose transporter (Hxt) as in the case of *S. cerevisiae* [36]. The utilization rate of Glc at 12 h was higher than that of Man, indicating the possibility that the K_m_ value for Man is larger than that for Glc [37]. The utilization rate of other sugars, especially at 12 h in the presence of Glc was low and the consumption was delayed compared to that in the absence of Glc. This also might be due not to glucose repression but to differences in the K_m_ values of transporters between Glc and other sugars and/or the limitation of ATP as an energy for transporters at high temperatures as mentioned above. Similar findings and discussion have been reported for *S. cerevisiae* [28]. Moreover, it is assumed that affinities of transporters for Gal, Xyl and Ara in CMUWF1–2 are weaker than that for Man because the presence of Man but not other sugars affected the Glc utilization pattern. There has no detailed report on sugar transport systems in *S. passalidarum*. The Xyl transporter system in *S. passalidaram* might be different from that in *S. cerevisiae* because the Hxt and galactose (Gal2) transporters in *S. cerevisiae*, which can uptake Xyl with a lower affinity than that for Glc, are strongly inhibited by Glc [38]. On the other hand, *S. passalidarum* was found to utilize Xyl even in the presence of Glc.

No effect of antimycin A on growth in YPXyl medium was found in CMUWF1–2 and *S. stipitis*. Both strains were able to grow in the presence of antimycin A, because xylose reductase (XR) in *S. stipitis* uses both NADPH and NADH as a cofactor with preference for NADPH [39] and XR in *S. passalidarum* also strongly prefers NADH [16]. On the other hand, XR in *K. marxianus* is highly specific for NADPH and its activity is weaker than those of most reported yeast XRs [40]. Therefore, it is likely that *K. marxianus* could not grow in the presence of antimycin A due to a redox imbalance.

## Conclusions

*S. passalidaram* CMUWF1–2, a new strain isolated from soil in Thailand, was found to have a high potential for conversion of various sugars to ethanol without significant glucose repression at a high temperature. The highest ethanol yield was found to be 0.43 g/g xylose with little accumulation of xylitol. The strain was more tolerant than *S. stipitis* to high concentrations of glucose and ethanol. This work provides valuable information on *S. passalidaram* CMUWF1–2, especially for its application to a high temperature fermentation with lignocellulosic biomass.

## Methods

### Strains and media

*S. stipitis* CBS5773, *K. marxianus* DMKU3–1042 and *S. cerevisiae* TISTR5606 used in this study were provided by Savitree Limtong (Kasetsart University, Bangkok, Thailand). YMXyl medium (3 g/l yeast extract, 3 g/l *w*/*v* malt extract, 5 g/l peptone, 10 g/l xylose and 7 g/l 1 N HCl) was used for screening of xylose-utilizing yeasts and isolation of xylose-fermenting yeasts. Cell cultures were carried out in YPXyl medium (10 g/l yeast extract, 20 g/l peptone and 20 g/l xylose), which was used for preparation of the inoculum. For investigating the utilization of an individual sugar, cells that were pre-cultured in YPD medium (10 g/l yeast extract, 20 g/l peptone and 20 g/l glucose) were inoculated into YP medium (10 g/l yeast extract and 20 g/l peptone) supplemented with 20 g/l of D-glucose (Glc), D-mannose (Man), D-galactose (Gal), D-xylose (Xyl) or L-arabinose (Ara) and cultured. These media were designated as YPD, YPMan, YPGal, YPXyl and YPAra, respectively. YP medium supplemented with 20 g/l Glc in combination with 20 g/l of Man, Gal, Xyl or Ara was used for experiments with mixed sugars, and these media were named YPDMan, YPDGal, YPDXyl and YPDAra, respectively. To examine the effect of antimycin A on xylose utilization, 5 μM antimycin A was added to Yeast Nitrogen base (YNB) supplemented with 20 g/l xylose.

### Isolation and analysis of growth and ethanol production of xylose-fermenting yeasts

Xylose-fermenting yeasts were isolated from natural samples such as soil, rotten fruit and decayed wood in Chiang Mai Province and Chiang Rai Province, Thailand. Screening was carried out using YMXyl medium. One gram of each sample was added to 9 ml of the medium and then 10-fold serially diluted and spread on YMXyl agar plates. After incubation at 30°C for 48 h, all yeast colonies that were able to grow on YMXyl plates were collected. After verifying their morphologies as yeast under a microscope, they were isolated on YPXyl agar plates. Isolated yeast strains were kept on YPD agar plates and stored at 4°C until used. Isolated xylose-assimilating yeast strains that grew well in YPXyl medium were further examined for their ability of ethanol production compared with that of *S. stipitis* and *K. marxianus*. For investigating ethanol production from Xyl, yeast strains were pre-cultivated in 3 ml of YPXyl medium for 18 h at 160 rpm, and the pre-culture was inoculated at 0.1% inoculum into a 300-ml Erlenmeyer flask containing 100 ml of a fresh YPXyl medium and cultured.

Additionally, the best xylose-fermenting yeast was selected to examine growth ability at different temperatures. Cells that had been cultivated in YPD medium for 18 h were collected and washed with sterile YP medium. Aliquots of cells containing about l × 10^7^cells/ml were 10-fold serially diluted and then spotted onto YPXyl agar plates and incubated at 30°C, 37°C, 40°C and 42°C for 48 h. *K. marxianus* and *S. stipitis* were used as control strains.

### Identification of a xylose-fermenting yeast

A yeast strain that produced the largest amount of ethanol from xylose was identified by nucleotide sequencing of the D1/D2 domain from the large-subunit (LSU) rRNA gene. Genomic DNA was extracted as described previously [41]. Forward primer NL-1 (5′-GCATATCAATAA GCGGAGGAAAAG-3′) and reverse primer NL-4 (5′-GGTCCGTGTTTCAAGACGG-3′) were used to generate the PCR product of the D1/D2 domain [42]. The PCR product was checked by agarose gel electrophoresis and purified by using a GF-1 AmbiClean Kit (Vivantis, Malaysia). The sequences of the PCR product were determined by 1st BASE (Selangor, Malaysia) and examined via a BLAST homology search [43]. The evolutionary history was inferred using the Neighbor-Joining method [44]. The evolutionary distances were computed using the Kimura 2-parameter method [45]. Evolutionary analyses were conducted using MEGA7 software [46].

### Cell growth and ethanol production from a single sugar or mixed sugars with glucose at different temperatures

For investigating ethanol production and ability of sugar utilization, yeast strains were pre-cultivated at 160 rpm for 18 h in 3 ml of YPD medium. The pre-culture was inoculated at 0.1% inoculum into a 300-ml Erlenmeyer flask containing 100 ml of a fresh YP medium including Glc, Man, Gal, Xyl or Ara or containing YPD medium supplemented with one of the other sugars. Cells were grown under a shaking condition (160 rpm) at 30 °C, 37 °C or 40 °C.

### Analytical methods

Cell biomass was determined turbidmetrically at 660 nm. Cell cultures were sampled and immediately subjected to centrifugation at 8000 *g* for 5 min. Sugar concentration in the supernatant was analyzed by high-performance liquid chromatography (HPLC) (Hitachi, Japan). A Gel pack column GL-C610-S (Hitachi, Japan) was used together with a refractive index detector (model L-2490, Hitachi) at 60 °C with 0.3 ml/min eluent of deionized water. Ethanol concentration in the supernatant was analyzed by either gas chromatography (GC) (model GC-2014, Shimadzu) or HPLC.

### Spot tests for testing effects of 2-deoxyglucose (2-DOG) on sugar utilization and for toleration of glucose and ethanol

Cells that had been pre-cultured in YPD medium for 18 h were collected, washed with a sterile YP medium, and suspended in the same medium. The suspension of cells containing about l × 10^7^ cells/ml was 10-fold serially diluted and spotted onto agar plates. For testing the effect of 2-DOG on Xyl utilization, YPXyl agar plates supplemented with 0.0, 0.01, 0.05, 0.10 and 0.20% (*w*/*v*) 2-DOG were used. For testing the effects of 2-DOG on utilization of other sugars and toleration of glucose and ethanol, YPGal, YPMan, YPAra and YPXyl agar plates supplemented or not supplemented with 0.01% or 0.2% 2-DOG were used. For testing glucose tolerance and ethanol tolerance, YP agar plates supplemented with 2, 20, 24, 28, 30, 35 and 40% (w/v) glucose and YPD agar plates supplemented with 0, 6, 8, 10 and 12% (*v*/v) ethanol were used. After spotting, plates were incubated at 30 °C for 48 h.

## Additional files

Additional file 1: **Table S1.** Xylose-utilizing yeasts isolated from natural samples and its sources. (PDF 357 kb)

Additional file 2: **Figure S1.** A phylogenetic tree of CMUWF1–2 isolated in this study and other strains published in databases was constructed. Numbers indicate percentages of bootstrap sampling, derived from 1000 samples. The numbers in parentheses are GenBank accession numbers. *Schizosaccharomyces pombe* NRRL Y-12796 was an outgroup in the analysis. (PDF 389 kb)

Additional file 3: **Table S2.** Parameters in YP medium containing a single sugar at various temperatures under a shaking condition (160 rpm). μ_x/s_, Specific growth rate; γ_s_, Specific sugar utilization rate; ±, S.D. from three independent experiments. (PDF 331 kb)

Additional file 4: **Table S3.** Parameters in YP medium containing mixed sugars with Glc at various temperatures under a shaking condition (160 rpm). μ_x/s_, Specific growth rate; γ_s_, Specific sugar utilization rate; ±, S.D. from three independent experiments. (PDF 255 kb)

Additional file 5: **Figure S2.** Effects of 2-DOG were examined on YPXyl plates containing various concentrations of 2-DOG. (PDF 411 kb)

Additional file 6: **Figure S3.** Effects of 2-DOG were examined on YPMan, YPGal, YPXyl, and YPAra plates with or without 0.01% 2-DOG. (PDF 367 kb)

Additional file 7: **Figure S4.** Glucose tolerance and ethanol tolerance of *S. passalidarum* CMUWF1–2. Glucose tolerance (**a)** and ethanol tolerance **(b**) of *S. passalidarum* CMUWF1–2 were compared with those of *K. marxianus* and *S. stipitis*. Data were reproduced by two independent experiments. (PDF 460 kb)

Additional file 8: **Table S4.** Comparison of xylose fermentation data in this study with those reported previously in *S. passalidarum* strains. ^a^cultivation times that required for reaching the maximum values or cultivation time of the end of the fermentation experiment.; ^b^Results for the best fermentation (fourth fed batch); WV, working volume, n; data not available or not tested; ND, not detected. (PDF 471 kb)

## Abbreviations

2-DOG: 2-deoxyglucose
GC: Gas chromatography
HPLC: High-performance liquid chromatography
