# Supplementary material for: Highly efficient conversion of xylose to ethanol without glucose repression by newly isolated thermotolerant Spathaspora passalidarum CMUWF1–2
Source: BMC Microbiol. 2018 Jul 13;18:73. doi: 10.1186/s12866-018-1218-4 (PMC6043994; doi:10.1186/s12866-018-1218-4)
Supplement: Supplementary file 1 — Table S1. Xylose-utilizing yeasts isolated from natural samples and its sources. (PDF 357 kb) [file 12866_2018_1218_MOESM1_ESM.pdf]

Table S1. Xylose-utilizing yeasts isolated from natural samples and its sources

No.	Isolates	Types of samples	Sources
1	CMUMY1-P1	Rotten pineapple	Muang District, Chiang Rai
2	CMUMY1-P3	Rotten pineapple	Muang District, Chiang Rai
3	CMUMY1-P4	Rotten pineapple	Muang District, Chiang Rai
4	CMUMY2-P1	Rotten pineapple	Muang District, Chiang Rai
5	CMUMY2-P2	Rotten pineapple	Muang District, Chiang Rai
6	CMUMY3-4	Decayed wood	Muang District, Chiang Rai
7	CMUMY3-5	Decayed wood	Muang District, Chiang Rai
8	CMUWF1-2	Soil	Mae Taeng District, Chiang Mai
9	CMUWF2-2	Soil	Mae Taeng District, Chiang Mai
10	CMULP5	Soil	Muang District, Chiang Mai
11	CMULP6	Soil	Muang District, Chiang Mai
12	CMULP7	Soil	Muang District, Chiang Mai
13	CMUF1	Soil	Muang District, Chiang Mai
