# Supplementary figures and images for: Highly efficient conversion of xylose to ethanol without glucose repression by newly isolated thermotolerant Spathaspora passalidarum CMUWF1–2
Source: BMC Microbiol. 2018 Jul 13;18:73. doi: 10.1186/s12866-018-1218-4 (PMC6043994; doi:10.1186/s12866-018-1218-4)

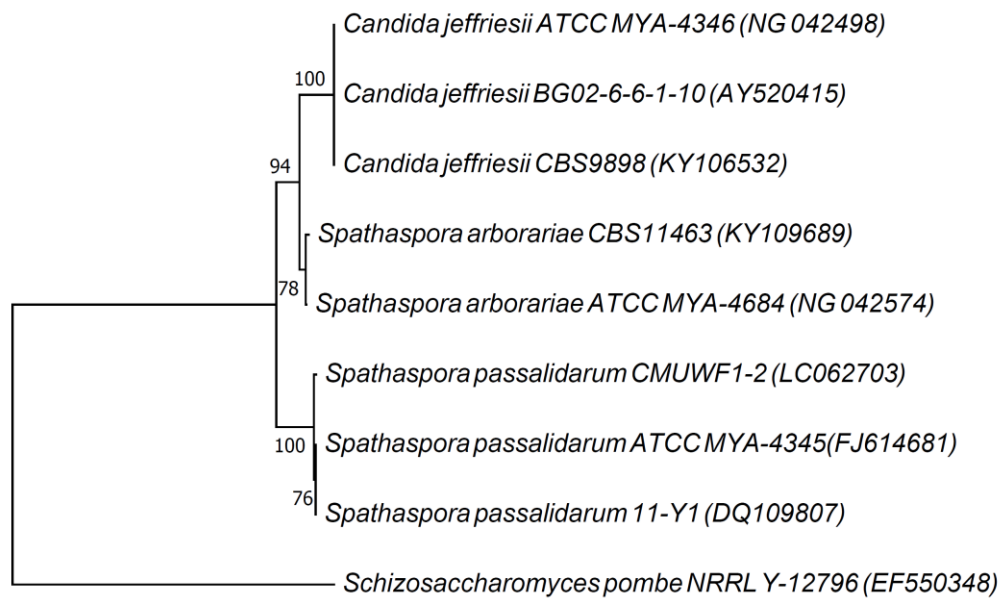

0.020

**Figure S1.**

Supplement: Supplementary file 2 — Figure S1. A phylogenetic tree of CMUWF1–2 isolated in this study and other strains published in databases was constructed. Numbers indicate percentages of bootstrap sampling, derived from 1000 samples. The numbers in parentheses are GenBank accession numbers. Schizosaccharomyces pombe NRRL Y-12796 was an outgroup in the analysis. (PDF 389 kb) [file 12866_2018_1218_MOESM2_ESM.pdf]

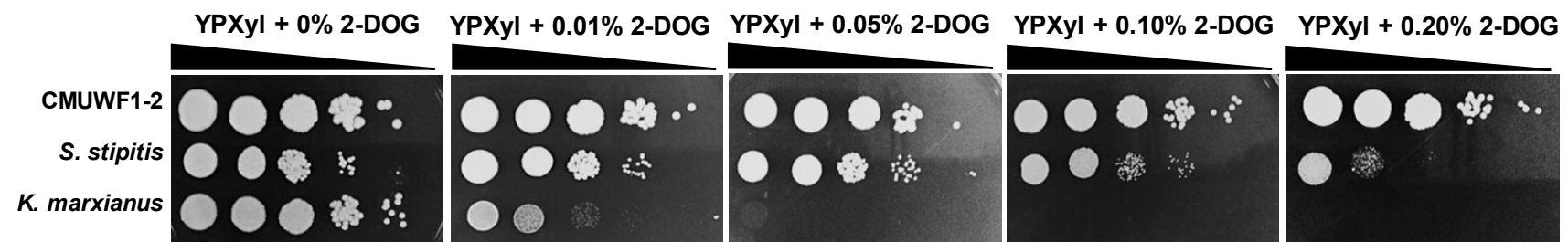

Figure S2.

Supplement: Supplementary file 5 — Figure S2. Effects of 2-DOG were examined on YPXyl plates containing various concentrations of 2-DOG. (PDF 411 kb) [file 12866_2018_1218_MOESM5_ESM.pdf]

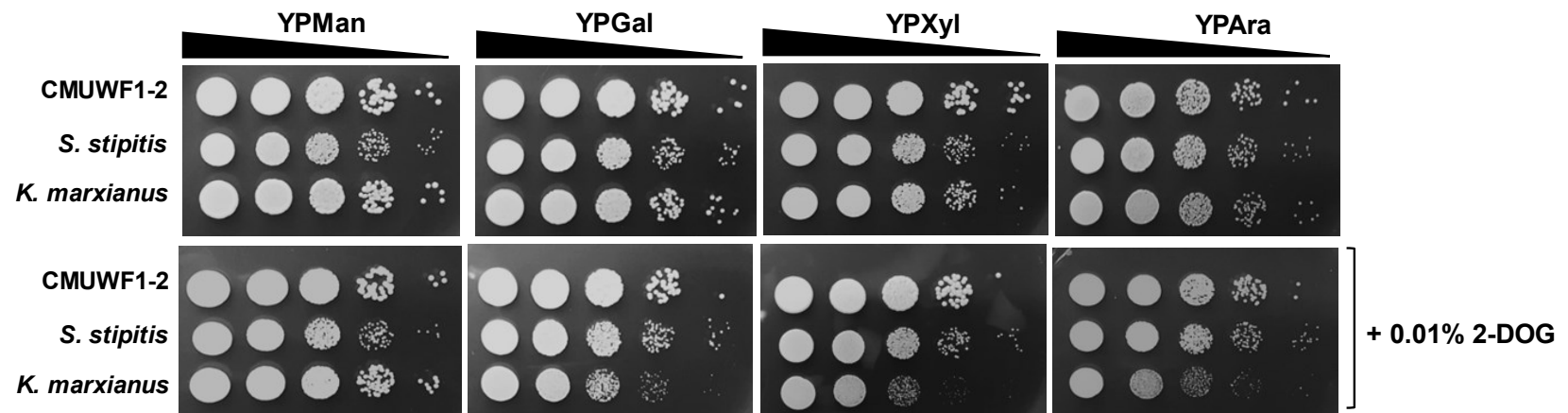

Figure S3.

Supplement: Supplementary file 6 — Figure S3. Effects of 2-DOG were examined on YPMan, YPGal, YPXyl, and YPAra plates with or without 0.01% 2-DOG. (PDF 367 kb) [file 12866_2018_1218_MOESM6_ESM.pdf]

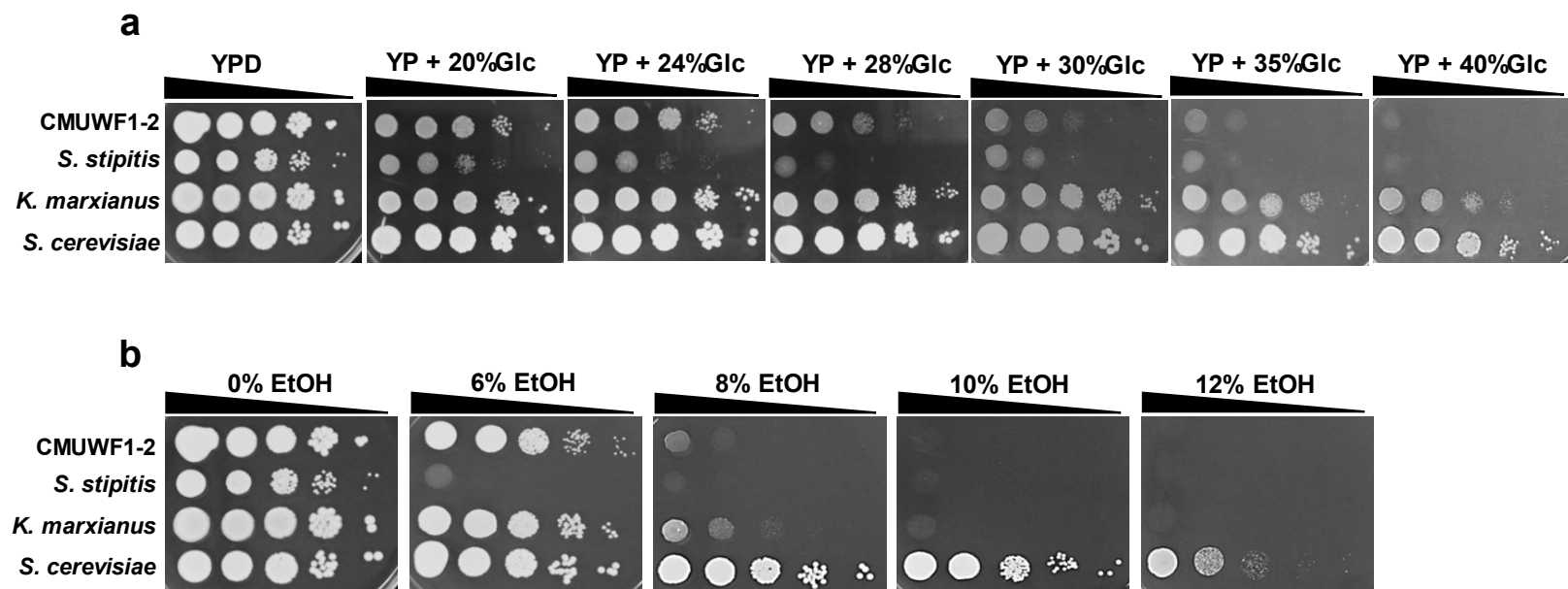

Figure S4.

Supplement: Supplementary file 7 — Figure S4. Glucose tolerance and ethanol tolerance of S. passalidarum CMUWF1–2. Glucose tolerance (a) and ethanol tolerance (b) of S. passalidarum CMUWF1–2 were compared with those of K. marxianus and S. stipitis. Data were reproduced by two independent experiments. (PDF 460 kb) [file 12866_2018_1218_MOESM7_ESM.pdf]
