# Supplementary material for: Highly efficient conversion of xylose to ethanol without glucose repression by newly isolated thermotolerant Spathaspora passalidarum CMUWF1–2
Source: BMC Microbiol. 2018 Jul 13;18:73. doi: 10.1186/s12866-018-1218-4 (PMC6043994; doi:10.1186/s12866-018-1218-4)
Supplement: Supplementary file 3 — Table S2. Parameters in YP medium containing a single sugar at various temperatures under a shaking condition (160 rpm). μx/s, Specific growth rate; γs, Specific sugar utilization rate; ±, S.D. from three independent experiments. (PDF 331 kb) [file 12866_2018_1218_MOESM3_ESM.pdf]

Table S2. Parameters in YP medium containing a single sugar at various temperatures under a shaking condition (160 rpm)

Medium	Temp (°C)	$\mu_{x/s}$ (h^{-1}) at 12 h	γ_s (g/l·h) at 12 h
YPD	30	1.09 ± 0.23	0.68 ± 0.28
	37	1.12 ± 0.09	0.71 ± 0.25
	40	0.30 ± 0.01	0.10 ± 0.08
YPMan	30	0.77 ± 0.26	0.34 ± 0.23
	37	0.83 ± 0.23	0.57 ± 0.26
	40	0.33 ± 0.13	0.14 ± 0.11
YPGal	30	0.64 ± 0.02	0.31 ± 0.23
	37	0.48 ± 0.30	0.26 ± 0.11
	40	0.18 ± 0.12	0.15 ± 0.08
YPXyl	30	0.47 ± 0.03	0.17 ± 0.15
	37	0.38 ± 0.21	0.15 ± 0.03
	40	0.13 ± 0.07	0.07 ± 0.05
YPAra	30	0.26 ± 0.02	0.03 ± 0.05
	37	0.17 ± 0.06	0.06 ± 0.06
	40	0.07 ± 0.03	0.01 ± 0.01

$\mu_{x/s}$, Specific growth rate; γ_s, Specific sugar utilization rate; ±, S.D. from three independent experiments
