# Supplementary material for: Highly efficient conversion of xylose to ethanol without glucose repression by newly isolated thermotolerant Spathaspora passalidarum CMUWF1–2
Source: BMC Microbiol. 2018 Jul 13;18:73. doi: 10.1186/s12866-018-1218-4 (PMC6043994; doi:10.1186/s12866-018-1218-4)
Supplement: Supplementary file 4 — Table S3. Parameters in YP medium containing mixed sugars with Glc at various temperatures under a shaking condition (160 rpm). μx/s, Specific growth rate; γs, Specific sugar utilization rate; ±, S.D. from three independent experiments. (PDF 255 kb) [file 12866_2018_1218_MOESM4_ESM.pdf]

Table S3. Parameters in YP medium containing mixed sugars with Glc at various temperatures under a shaking condition (160 rpm)

Medium	Temp (°C)	$\mu_{x/s}$ (h^{-1}) at 12 h	γ_s (g/l h) at 12 h	
YPDMan	30	1.08 ± 0.25	Glc	0.51 ± 0.12
			Man	0.10 ± 0.09
	37	1.00 ± 0.02	Glc	0.59 ± 0.04
			Man	0.23 ± 0.08
	40	0.20 ± 0.10	Glc	0.03 ± 0.07
			Man	0.02 ± 0.02
YPDGal	30	1.07 ± 0.20	Glc	0.72 ± 0.15
			Gal	0.01 ± 0.05
	37	1.03 ± 0.07	Glc	0.67 ± 0.11
			Gal	0.01 ± 0.01
	40	0.19 ± 0.10	Glc	0.08 ± 0.06
			Gal	0.01 ± 0.01
YPDXyl	30	1.06 ± 0.39	Glc	0.72 ± 0.31
			Xyl	0.02 ± 0.02
	37	0.95 ± 0.06	Glc	0.76 ± 0.10
			Xyl	0.08 ± 0.07
	40	0.19 ± 0.09	Glc	0.13 ± 0.09
			Xyl	0.03 ± 0.02
YPDAra	30	1.02 ± 0.35	Glc	0.75 ± 0.22
			Ara	0.08 ± 0.09
	37	1.07 ± 0.04	Glc	0.75 ± 0.15
			Ara	0.00 ± 0.04
	40	0.22 ± 0.12	Glc	0.14 ± 0.03
			Ara	0.01 ± 0.01

$\mu_{x/s}$, Specific growth rate; γ_s, Specific sugar utilization rate; ±, S.D. from three independent experiments
