# Supplementary material for: Highly efficient conversion of xylose to ethanol without glucose repression by newly isolated thermotolerant Spathaspora passalidarum CMUWF1–2
Source: BMC Microbiol. 2018 Jul 13;18:73. doi: 10.1186/s12866-018-1218-4 (PMC6043994; doi:10.1186/s12866-018-1218-4)
Supplement: Supplementary file 8 — Table S4. Comparison of xylose fermentation data in this study with those reported previously in S. passalidarum strains. acultivation times that required for reaching the maximum values or cultivation time of the end of the fermentation experiment.; bResults for the best fermentation (fourth fed batch); WV, working volume, n; data not available or not tested; ND, not detected. (PDF 471 kb) [file 12866_2018_1218_MOESM8_ESM.pdf]

Table S4. Comparison of xylose fermentation data in this study with those reported previously in *S. passalidarum* strains.

Strains	Temp. (°C)	Conditions	Substrate conc. (g/l)	Time (h)[a]	EtOH production (g/l)	EtOH Yield (g/g)	Xylitol production (g/l)	Reference
S. passalidarum CMUWF1-2	30	Flask (WV 100 ml in 300 ml flask, 160 rpm)	(20) xylose	24	8.36 ± 0.22	0.43 ± 0.03	0.03	This study
			(20) glucose + (20) xylose	24	16.5 ± 0.75	0.42 ± 0.03	0.51	
	37		(20) xylose	24	6.77 ± 0.38	0.40 ± 0.04	0.19	
			(20) glucose + (20) xylose	36	16.0 ± 0.44	0.39 ± 0.02	0.87	
	40		(20) xylose	48	3.90 ± 2.46	0.20 ± 0.12	0.43	
			(20) glucose + (20) xylose	60	11.7 ± 1.57	0.33 ± 0.04	0.37	
S. passalidarum ATCC MYA-4345	25	Bioreactor (WV 2,000 ml)	(40) xylose + (60) cellobiose	68	~38.0	0.43	n	[1]
			(40) glucose + (40) xylose+ (20) cellobiose	68	~40.0	0.42	n	
	32	Anaerobic vials (WV 50 ml in 100 ml, 120 rpm)	(30) xylose + (30) glucose	96	~28.0	n	n	[2]
		Flask (WV 50 ml in 100 ml flask, 120 rpm)	(30) xylose + (30) glucose	24	~25.0	n	n	
	25	Flask (WV 70 ml in 125 ml flask, 110 rpm)	(150) xylose	120	~56.0	0.45	0.80 – 1.40	[3]
			(120) xylose + (30) glucose	120	58.3	0.43	2.90 – 3.80	
	30	Flask (WV 100 ml in 250 ml flask with cotton plugged)	(40–50) xylose	18	20.3 ± 0.20	0.48	0.60 ± 0.00	[4]
		Flask (WV 100 ml in 100 ml flask with rubber plugged and needle)	(40–50) xylose	24	20.5 ± 0.10	0.48	1.00 ± 0.20	
	27	Bioreactor-sequential fed batch cell recycle (WV 700 ml)[b]	Sugarcane bagasse hydrolysate (42.9) glucose + (14.9) xylose	24	23.3	0.46	0.67	[5]
S. passalidarum UFMG-HMD-1.1, UFMG-HMD-1.3, UFMG-HMD-2.1, UFMG-HMD-10.2, UFMG-HMD-14.1, UFMG-HMD-16.2	30	Flask: (WV 50 mL in 125 ml flasks	(50) xylose	24	15.0 - 18.0	0.31 - 0.37	1.00 - 2.20	[6]
S. passalidarum UFMG-CM-Y469	30	Flask (WV 100 ml in 250 ml flask with cotton plugged)	(40–50) xylose	18	20.2 ± 0.10	0.47	1.10 ± 0.00	[4]
		Flask (WV 100 ml in 100 ml flask with rubber plugged and needle)	(40–50) xylose	24	20.5 ± 0.60	0.48	1.40 ± 0.01	
S. passalidarum P16-1.1, G13-2.1, G13-3.8, G14-1.8, G14-2.2 and G18-3.7	28	Flask (50 ml WV in 125 ml flask, 120 rpm)	(40) xylose	30	17.9 - 18.9	0.42 - 0.45	ND	[7]

[a] cultivation times that required for reaching the maximum values or cultivation time of the end of the fermentation experiment.; [b] Results for the best fermentation (fourth fed batch); WV, working volume, n; data not available or not tested; ND, not detected

1. Long TM, Su YK, Headman J, Higbee A, Willis LB, Jeffries TW: Cofermentation of glucose, xylose, and cellobiose by the beetle-associated yeast *Spathaspora passalidarum*. Appl Environ Microbiol 2012, 78(16): 5492-5500.
2. Hou X: Anaerobic xylose fermentation by *Spathaspora passalidarum*. Appl Microbiol Biotechnol 2012, 94(1):205-214.
3. Su YK, Willis LB, Jeffries TW: Effects of aeration on growth, ethanol and polyol accumulation by *Spathaspora passalidarum* NRRL Y-27907 and *Scheffersomyces stipitis* NRRL Y-7124. Biotechnol Bioeng 2015, 112(3): 457-469.
4. Cadete RM, de Las Heras AM, Sandstrom AG, Ferreira C, Girio F, Gorwa-Grauslund MF, Rosa CA, Fonseca C: Exploring xylose metabolism in *Spathaspora* species: XYL1.2 from *Spathaspora passalidarum* as the key for efficient anaerobic xylose fermentation in metabolic engineered *Saccharomyces cerevisiae*. Biotechnol Biofuels 2016, 9: 167.
5. Nakanishi SC, Soares LB, Biazi LE, Nascimento VM, Costa AC, Rocha GJM, Ienczak JL: Fermentation strategy for second generation ethanol production from sugarcane bagasse hydrolyzate by *Spathaspora passalidarum* and *Scheffersomyces stipitis*. Biotechnol Bioeng. 2017, 114(10): 2211-2221.
6. Cadete RM, Melo MA, Dussan KJ, Rodrigues RC, Silva SS, Zilli JE, Vital MJ, Gomes FC, Lachance MA, Rosa CA: Diversity and physiological characterization of D-xylose-fermenting yeasts isolated from the Brazilian Amazonian Forest. PLoS One 2012, 7 (8):e43135.
7. Souza GdF, Leite a, Valentim LTCN, Nogueira SRP, Abegg MA: Efficient production of second generation ethanol and xylitol by yeasts from Amazonian beetles (Coleoptera) and their galleries. Afr J Microbiol Res. 2017, 11(20): 814-824.
